# Metabolic Dynamics of Ecosystems Realizing Steady Log-Uniform Distributions: The Case of Commodities in Shops

**DOI:** 10.3390/e22030267

**Published:** 2020-02-26

**Authors:** Gen Sakoda, Hideki Takayasu, Misako Takayasu

**Affiliations:** 1 Department of Mathematical and Computing Sciences, School of Computing, Tokyo Institute of Technology, 4259 Nagatsuta-cho, Midori-ku, Yokohama 226-8502, Japan; sakoda.g.aa@m.titech.ac.jp; 2 Sony Computer Science Laboratories, 3-14-13 Higashi-Gotanda, Shinagawa-ku, Tokyo 141-0022, Japan; takayasu@csl.sony.co.jp; 3 Institute of Innovative Research, Tokyo Institute of Technology, 4259 Nagatsuta-cho, Midori-ku, Yokohama 226-8502, Japan

**Keywords:** log-uniform, metabolism, ecosystem, random multiplicative diffusion, point-of-sales, econophysics

## Abstract

From the viewpoint of statistical physics, ecosystems in the real world are very attractive targets of research as examples of far-from thermal equilibrium systems where various kinds of components are coming in and out continuously while keeping the whole systems quasi-stationary. As a fortunate example of a fully-observable ecosystem, we analyzed the comprehensive data of convenience stores where approximately 5% of the commodity species is replaced by new ones daily. The share of stores for each species fluctuates significantly; however, the entire distribution of shares is fairly stationary and follows the log-uniform distribution, that is, the power law distribution with exponent 0. We introduce an empirical time evolution model of shares and firstly deduce that the key mechanism of realizing this stationary distribution is random multiplicative diffusion in finite size spaces. Our model based on the general stochastic process is expected to be applicable to various dynamic systems, especially complex systems with highly nonlinear interactions.

## 1. Introduction

Power law distributions and their physical mechanisms have attracted the attention of physicists for more than a century [1]. In 1916, Smoluchowski considered a power law distribution for a mass of colloidal particles [2], and subsequently it was clarified that the steady-state power law was generally realized via the irreversible coagulation process under a continuous injection of finer mass particles [3,4,5,6,7,8]. Generally, power laws are also observed at the critical point of phase transitions, such as the cluster size distribution of a percolation system [9]. The concept of self-organized criticality [10] illustrates that some systems automatically tune the underlying control parameters to sit at the critical point. The power-law behaviors observed in avalanches [10], solar flares [11], earthquakes [12], and biological evolution [13] are categorized as such systems. Moreover, physicists have expanded the research targets to include economic and social phenomena. Consequently, power law distributions have been observed in financial market price changes [14], income distribution of companies [15], link distribution of the network structure in business relationships [16], etc.

Several studies have also employed mathematical approaches for power law distributions. In 1925, Yule introduced the rich-get-richer mechanism to explain the power law distribution in the sizes of biological genera [17]. This mechanism also explains power law behaviors in other phenomena such as word frequency [18] and the distribution of links in complex networks [19]. In the 1930s, Levy generalized the central limit theorem and showed that power law distributions are stable under the summation of independent random variables [20]. Random multiplicative process or the Langevin equation with a randomly changing coefficient is also known to produce power law distributions [21,22].

Although several studies have focused on power law distributions, one particular case has attracted lesser attention: the power law distribution with the power exponent 0, which is also known as the log-uniform distribution, as the probability density for the variable becomes flat after taking logarithm [23]. Log-uniform distribution is not only a theoretical distribution for statistical consideration or an assumption in a computer simulation [24], but also an actual distribution observed in natural and social phenomena. Concretely, in astrophysics, the class I protostellar binary separation distribution is considered to fit a log-uniform distribution [25]. In the field of earth science, distributions of curvilinear inter distances between orogenic gold deposits along the Archean fault zones are found to follow a log-uniform distribution [26]. In the case of human activities, the distribution of user-estimate computing time for each posted job is reported to follow a log-uniform distribution [27]. However, in contrast to other power law distributions with non-zero exponents, reports on log-uniform distributions in the real world are scarce, and its origins remain unclear.

In this paper, we report that a log-uniform distribution is identified in the commodity ecosystem of convenience stores through big-data analysis of the Point-Of-Sales (POS) data. Thereafter, we introduce a mathematical model and elucidate the general dynamics of the log-uniform distribution in the ecosystem.

## 2. Materials

Our POS data are obtained from 326 chain stores of a convenience store company named Seven-Eleven Japan Co., Ltd. These data contain the record of every purchase at cash registers, daily stocks, and daily disposals, for each commodity and shop for a period of 153 days, from June 2010 to October 2010. The 326 stores are located in Kanagawa and Yamaguchi Prefecture of Japan. These stores sell extensive ranges of commodity species, including daily food, fast food, newspapers, magazines, stationeries, cigarettes, kitchen utensils, liquors, batteries, and even pet foods. The average floor space per store is about 123 square meters. The average operating revenue per store in 2010 was about 42 million yen [28].

## 3. Results

### 3.1. Metabolism of Commodity Species

**Figure 1:**
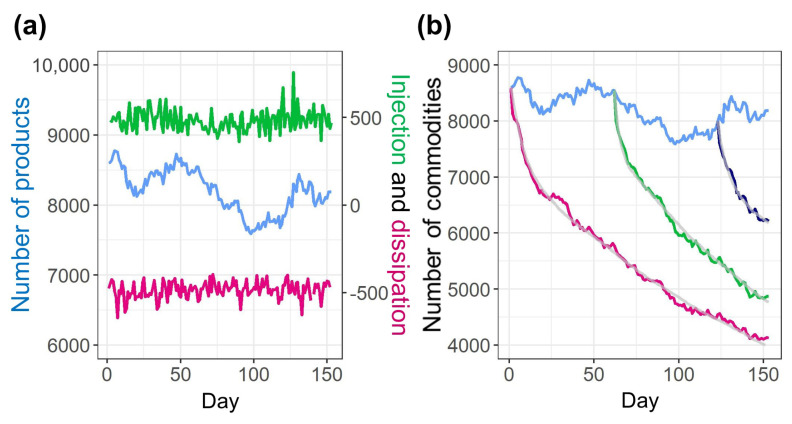
Metabolism of the commodity ecosystem. (**a**) Injection and dissipation of commodity species. The blue line denotes the total amount of commodity species, green line denotes the injection, and red line denotes the dissipation. (**b**) Dissipation trend of commodity species. The blue line denotes the total number of commodity species. The red, green, and navy lines denote the dissipations of commodity species that exist on day 1, 62, and 123, respectively. Each gray line on the red, green, and navy lines denotes the simulated dissipation with the exponential mixture distribution.

The POS data clarifies the metabolism of commodity species. Figure 1a depicts the daily injection and dissipation of commodity species. The blue line denotes the total amount of commodity species, the green line indicates the injection, and the red line indicates the dissipation. Although the total amount of commodity species fluctuate around 8200, there is a steady injection and dissipation of approximately 500 daily. The injection and dissipation for each commodity occurs several times; hence, the unique commodity species amount to 21,037 during the period of 153 days. Figure 1b illustrates the daily dissipation trend of commodity species, which indicates the lifetime of commodity species. The blue line indicates the total number of commodity species, and the red line indicates the dissipation of commodity species on day 1 (1 June 2010). The commodity species decrease from 8602 on day 1 to 4129 on day 153 (31 October 2010). The green and navy lines denote examples of the dissipation of commodity species that exist on day 62 (1 August 2010) and day 123 (1 October 2010), respectively. The dissipation trends decrease exponentially and are characterized as a mixture of short lifetime in the early stages and long lifetime thereafter. We adopted the exponential mixture distribution to approximate this lifetime.

(1) $$N\left(t\right)=a_{1}\left(R_{1}exp\left(-\left(t-1\right)/\mu_{1}\right)+\left(1-R_{1}\right)exp\left(-\left(t-1\right)/\mu_{2}\right)\right)$$

where N(t) is the number of commodities at day *t*, $a_{1}$ is the number of commodities at day 1, $\mu_{1}$ and $\mu_{2}$ are the mean values of the two exponential distributions, and $R_{1}$ is the mixture ratio of the two exponential distribution. The parameters of the mixture distribution are determined as follows:

The 25 dissipation trends—1st, 6th, 11th, 16th, 21st, and 26th of each month from June to September, and October 1st—are used to determine the parameters. Random number simulations involving the exponential mixture distribution are performed to determine the lifetime for each commodity species. The simulated and the original dissipation trends are compared, and the parameters with the least mean squared error are adopted for each trend. The median of the parameters are $\mu_{1}=3$, $\mu_{2}=205$, and $R_{1}=0.12$. The interquartile ranges (IQRs) are $\mu_{1}=2$, $\mu_{2}=36.7$, and $R_{1}=0.037$. Each gray line on the red, green, navy lines in Figure 1b denotes the example of the simulated dissipation.

### 3.2. Log-Uniform Distribution

**Figure 2:**
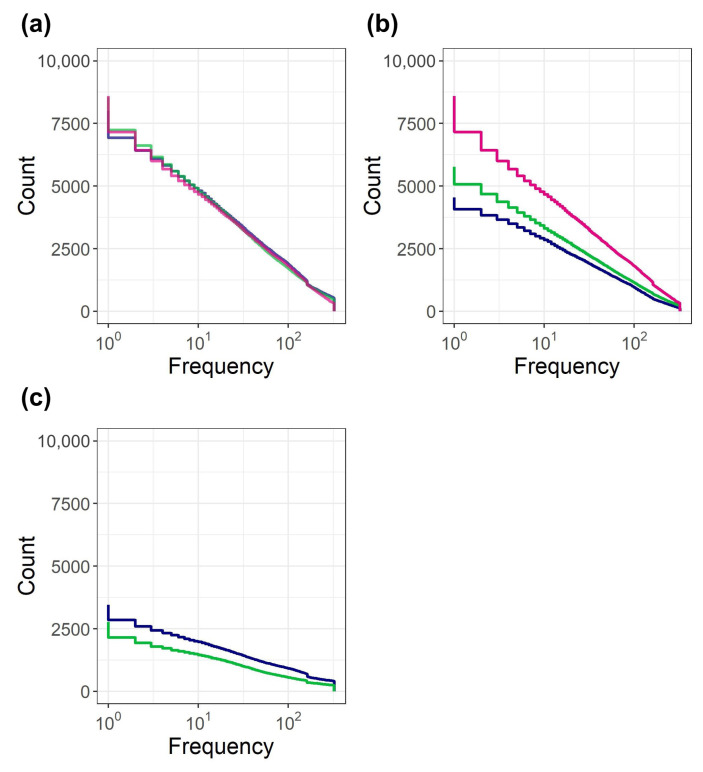
Log-uniform distribution observed in the frequency of shops to which each commodity species belongs. (**a**) Cumulative frequency distribution. (**b**) Transition of the log-uniform distribution on day 1. (**c**) Distribution of the frequency of shops to which each commodity species injected after day 1 belongs. In (**a**–**c**), the red, green, and navy lines denote the distributions on day 1, 62, and 123, respectively.

Generally, the positive variable *X* follows a log-uniform distribution in the case that log(X) follows a uniform distribution. Figure 2a depicts the log-uniform distribution observed in the commodity ecosystem. In this figure, the frequency of shops to which each commodity species belongs is aggregated without any pretreatment, and indicated in the cumulative frequency distribution. Since the total number of species in the distribution is clarified, the cumulative frequency distribution is used instead of the cumulative distribution function of which maximum value is normalized to one. As only the horizontal axis is logarithmic, linear dependency indicates that the data follows a log-uniform distribution. The red, green, and navy lines denote the distributions on day 1, 62, and 123, respectively. The linear dependency is generally independent of time.

Figure 2b presents the transition of the log-uniform distribution on day 1. The red, green, and navy lines denote the distributions on day 1, 62, and 123, respectively. Although the total amount of species on day 1 gradually decreases, the linear dependency of the distribution is generally maintained after the transition with dissipation. Figure 2c depicts the distribution of the frequency of shops to which each commodity species injected after day 1 belongs. The green and navy lines denote the distributions on day 62 and 123, respectively. These lines generally depict the linear dependencies. The injected commodity species also follow log-uniform distributions.

### 3.3. Dynamics of the Log-Uniform Distribution

**Figure 3:**
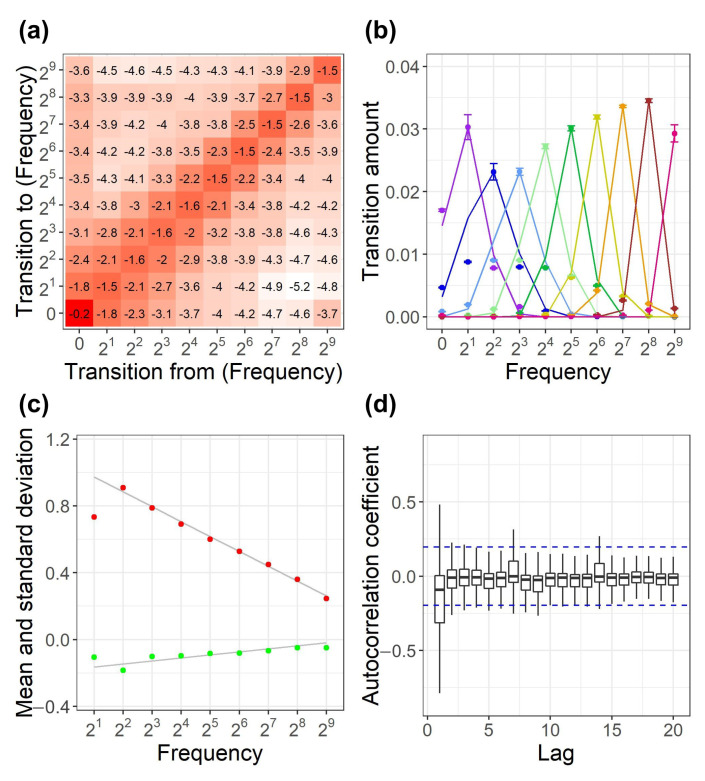
Transition of the frequency of shops to which each commodity species belongs. (**a**) Transition amount between exponential bins. The numbers in the heatmap are the transition amount in the $log_{10}$ scale. (**b**) Transition amount from each bin to the adjacent bins. Each color plot corresponds to the source bin: purple denotes $2^{1}$, blue denotes $2^{2}$, light blue denotes $2^{3}$, light green denotes $2^{4}$, green denotes $2^{5}$, yellow denotes $2^{6}$, orange denotes $2^{7}$, brown denotes $2^{8}$, and red denotes $2^{9}$. Each color line indicates the log-normal distribution estimated via the maximum likelihood estimation. (**c**) Coefficient of the log-normal distribution. The red plot denotes the standard deviation log${}_{2}\left(\sigma \right)$, and the green plot denotes the mean log${}_{2}\left(\mu \right)$. Each gray line denotes the linear regression line. (**d**) Box-and-whisker plot of the autocorrelation coefficient of the multiple coefficient b(t). The blue dashed lines denote 95% confidence intervals.

To clarify the mechanism of log-uniform distribution, we examined the daily transition of the distribution in detail. Let x(t) be the frequency of shops to which a commodity species belongs on day *t*, x(t) was grouped in exponential bins such as $\left[1,2\right),\left[2,2^{2}\right),\left[2^{2},2^{3}\right),\ldots$, and the distribution of diffusion for each bin was examined. Figure 3a depicts the amount of transition between each bin. The horizontal axis denotes the source bins, and the vertical axis indicates the destination bin, where $2^{N}$ corresponds to $\left[2^{N-1},2^{N}\right)$, and 0 refers to the amount of uninjected or dissipated commodity species. The total amount is normalized to 1. The numbers in the heatmap illustrate the amount of transition in the $log_{10}$ scale. The transition of each bin mainly occurs to the adjacent bins, which is typical for the diffusion mechanism.

Figure 3b shows the transition amount from each bin to adjacent bins. Each plot color corresponds to the source bin. Specifically, purple denotes $2^{1}$, blue denotes $2^{2}$, light blue denotes $2^{3}$, light green denotes $2^{4}$, green denotes $2^{5}$, yellow denotes $2^{6}$, orange denotes $2^{7}$, brown denotes $2^{8}$, and red denotes $2^{9}$. The amount of transition to a bin which is apart from a source bin is relatively smaller than that of the adjacent bins. Neglecting the bins which are apart from a source bin over 2 bins, the transition is generally approximated with the log-normal distribution. Each colored line in Figure 3b corresponds to the log-normal distribution, which is estimated via the maximum likelihood estimation. Figure 3c depicts the coefficient of the log-normal distribution for each bin. The red plot denotes the standard deviation log${}_{2}\left(\sigma \right)$, and the green plot indicates the mean log${}_{2}\left(\mu \right)$. Each gray line on the red and green plots denotes the linear regression line obtained using the data between $2^{2}$ and $2^{8}$. Here, the data at the boundaries $2^{1}$ and $2^{9}$ are excluded because half of the distribution are not observed. The regression functions are log${}_{2}\left(\sigma \right)=-0.089\cdot$ log${}_{2}\left(x\left(t\right)\right)+1.062$ and log${}_{2}\left(\mu \right)=0.018\cdot$ log${}_{2}\left(x\left(t\right)\right)-0.182$.

We examined the time dependecy of the distribution shown in Figure 3b by analyzing the deviation of the six distributions obtained using the transition data accumulated each month. The error bar for each plot in Figure 3b is 1 σ of the deviation. Generally, the diffusion follows a log-normal distribution independent of time.

The diffusion distribution implies that x(t) follows the random multiplicative diffusion, because the transition between exponential bins corresponds to the multiplication with the exponential base. Specifically,

(2) x(t+1)=b(t)x(t)

where b(t) is the multiple coefficient following a log-normal distribution.

To verify the randomness of the multiplication, we checked the autocorrelation of the multiple coefficient b(t). Figure 3d presents the box-and-whisker plot of the autocorrelation coefficient of b(t). The box for each lag represents the IQR, and the horizontal line in the box indicates the median. The whisker ranges from the maximum to the minimum values for 1.5 times of IQR from the upper and lower bound of the box. The blue dashed lines at ±0.196 denote the 95% confidence intervals under the null hypothesis that the autocorrelation coefficient is zero. The absolute values of the autocorrelation are generally smaller than the blue line except for the 42% of lag 1. Generally, b(t) is approximated to be random.

### 3.4. Random Multiplicative Diffusion Simulation

**Figure 4:**
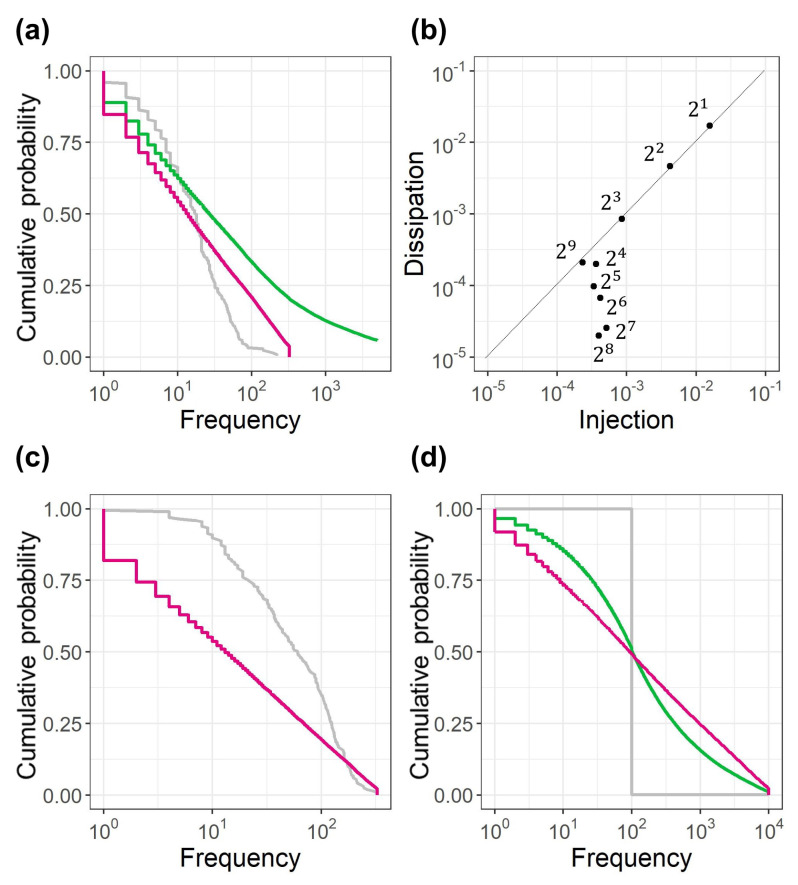
Results of the random multiplicative diffusion simulation. (**a**) The gray line denotes the result with one initial value x(1)=50, lifetime 205, and b(t) with log-normal distribution (log${}_{2}\left(\sigma \right)=0.5$ and log${}_{2}\left(\mu \right)=0$). The green line denotes the result with the 500 initial values sampled from the log-uniform distribution on day 1 shown in Figure 2a. The lifetimes are random numbers from the exponential mixture distribution ($\mu_{1}=3$, $\mu_{2}=205$, and $R_{1}=0.12$). The red line denotes the result with the same conditions as green line except for the closed boundary [1, 326]. (**b**) Injection and dissipation for each bin. (**c**) Result for the condition with the injection and dissipation in (**b**), standard deviation and mean of the log-normal distribution of b(t) shown in Figure 3c, and boundary [1, 326]. The gray line denotes the initial 500 values sampled from the log-uniform distribution on day 1 and dissipated proportional to the dissipation ratio shown in (**b**). The red line denotes the result with the lifetime values generated by the exponential mixture distribution ($\mu_{1}=3$, $\mu_{2}=205$, and $R_{1}=0.12$). (**d**) Result obtained with one value for the initial 1000 values, b(t) with log-normal distribution (log${}_{2}\left(\sigma \right)=0.5$ and log${}_{2}\left(\mu \right)=0$), and boundary [1, 10,000]. The gray line denotes the initial distribution, green line denotes the result for the lifetime 500, and red line denotes the result for the lifetime 1000.

Figure 4a presents a result of random multiplicative diffusion simulation. In the random multiplicative diffusion simulation, the time evolution of x(t) is determined by Equation (2). Specifically, x(t+1) is determined with the multiplication of x(t) and a random number b(t). Here, b(t) is assumed to follow the log-normal distribution with log${}_{2}\left(\sigma \right)=0.5$ and log${}_{2}\left(\mu \right)=0$ for simplicity. The gray line denotes the distribution obtained via 205 random multiplications with an initial value of x(1)=50. Note that 205 corresponds to the estimated lifetime $\mu_{2}$. As expected, a log-normal distribution is obtained because the distribution is produced using random walks in log-space. The green line indicates the distribution obtained with random multiplications of 500 initial values, which are sampled without replacement from the log-uniform distribution on day 1 shown in Figure 2a. The lifetime of random multiplication for each initial value is generated using random number simulations, assuming the exponential mixture distribution with $\mu_{1}=3$, $\mu_{2}=205$, and $R_{1}=0.12$. Although a linear dependency appears around the frequency from $10^{1}$ to $10^{2}$, the larger values diverge to infinity. The red line indicates the result of the simulation, assuming a closed boundary [1,326]. Note that the maximum value corresponds to the total number of shops in the POS data, and the minimum value is the smallest value while existing. Except for the boundary, the simulation conditions are the same as those for the green line. The divergence to infinity is suppressed. The assumption of the closed boundary is consistent with the system. Figure 4b depicts the injection and dissipation for each exponential bin, which is extracted from Figure 3a. Generally, the injection and dissipation are balanced at bin $2^{1}$ and $2^{9}$, which correspond to the lower and upper boundaries, respectively. As the dissipation is compensated with the injection at the boundary, diffusion across the boundary is regarded to be blocked at the boundary. Figure 4b provides additional information indicating that the injection and dissipation are also balanced at the $2^{2}$ and $2^{3}$, the injection amounts are generally log-uniform between $2^{4}$ and $2^{8}$, and dissipation amounts between $2^{4}$ and $2^{8}$ gradually decrease with increasing frequency.

Figure 4c depicts the result of the random multiplicative diffusion simulation, which considers the injection and dissipation for each bin shown in Figure 4b. In this simulation, the standard deviation and the mean of the log-normal distribution of b(t) for each x(t) are calculated using the regression functions obtained in Figure 3c. The boundary is set to be [1,326]. The gray line is the initial distribution, namely, 500 initial values are sampled without replacement from the log-uniform distribution on day 1. The initial values are dissipated proportional to the dissipation ratio to the injection for each bin which is shown in Figure 4b. The lifetime values are the random numbers generated by the exponential mixture distribution with $\mu_{1}=3$, $\mu_{2}=205$, and $R_{1}=0.12$. The red line indicates the result of random multiplicative diffusion simulation. The formation of a log-uniform distribution with the actual condition of the diffusion b(t), injection and dissipation, and the lifetime are verified.

To clarify the conditions for forming a log-uniform distribution, an additional random multiplicative diffusion simulation is performed. The initial 1000 values are not sampled from the log-uniform distribution, but assumed to be only one value 1000. The boundary is set to be [1, 10,000], which is broader and more general compared to the boundary [1,326] for the POS data. b(t) is assumed to be the log-normal distribution with log${}_{2}\left(\sigma \right)=0.5$ and log${}_{2}\left(\mu \right)=0$ for simplicity. Figure 4d presents the result of the random multiplicative diffusion simulation. The gray line indicates the initial distribution, the green line indicates the result with the lifetime 500, and the red line indicates that with lifetime 1000 for each initial value. The convergence to the log-uniform distribution is presented by the red line.

## 4. Conclusions

We conclude that if the lifetime is sufficiently long for values to spread the closed space, initial values following a log-uniform distribution are not necessary to form a log-uniform distribution. In the case that the lifetime is insufficient, as in the commodity ecosystem, the mixture of log-normal distributions with spreading initial values realize the log-uniform distribution. Random multiplicative diffusion in the logarithmic closed space is the necessary condition for each case. In the future researches, log-uniform distributions reported in fields such as astrophysics [25], earth science [26], and human activities [27] should be examined to determine if the diffusion mechanism discussed in this study is applicable. Our model based on the general stochastic process is expected to be applied to the consideration of the scaling laws in various natural and social phenomena.

## References

[1] Newman M.E.J. (2005). Power laws, Pareto distributions and Zipf’s law. Contemp. Phys..

[2] Smoluchowski M.V. (1916). Drei vortrage uber diffusion, Brownsche beregund und koagulation von kolloidteilchen. Zeitschrift für Physik.

[3] Takayasu H. (1989). Steady-state distribution of generalized aggregation system with injection. Phys. Rev. Lett..

[4] Takayasu M. (1992). Universal power law observed in an exponentially growing particle system. Phys. Rev. A..

[5] Takayasu H., Taguchi Y.-h. (1993). Non-Gaussian distribution in random advection dynamics. Phys. Rev. Lett..

[6] Takayasu M., Takayasu H., Taguchi Y.-h. (1994). Non-Gaussian distribution in random transport dynamics. Int. J. Mod. Phys. B.

[7] Krapivsky P.L., Redner S. (1996). Transitional aggregation kinetics in dry and damp environments. Phys. Rev. E.

[8] Friedlander S.K. (2000). Smoke, Dust, and Haze: Fundamentals of Aerosol Dynamics.

[9] Stauffer D., Aharony A. (1994). Introduction to Percolation Theory Rev..

[10] Bak P., Tang C., Wiesenfeld K. (1987). Self-organized criticality: An explanation of the 1/f noise. Phys. Rev. Lett..

[11] Lu E.T., Hamilton R.J. (1991). Avalanches and the Distribution of Solar Flares. Astrophys. J..

[12] Olami Z., Feder H.J.S., Christensen K. (1992). Self-organized criticality in a continuous, nonconservative cellular automaton modeling earthquakes. Phys. Rev. Lett..

[13] Bak P., Sneppen K. (1993). Punctuated equilibrium and criticality in a simple model of evolution. Phys. Rev. Lett..

[14] Mizuno T., Kurihara S., Takayasu M., Takayasu H. (2003). Analysis of high-resolution foreign exchange data of USD-JPY for 13 years. Physica A.

[15] Okuyama K., Takayasu M., Takayasu H. (1999). Zipf’s law in income distribution of companies. Physica A.

[16] Miura W., Takayasu H., Takayasu M. (2012). Effect of Coagulation of Nodes in an Evolving Complex Network. Phys. Rev. Lett..

[17] Yule G.U. (1925). II.—A mathematical theory of evolution, based on the conclusions of Dr. J. C. Willis, F. R. S. Phil. Trans. R. Soc. Lond. B.

[18] Simon H.A. (1955). On a Class of Skew Distribution Functions. Biometrika.

[19] Barabási R., Albert A. (1999). Emergence of Scaling in Random Networks. Science.

[20] Feller W. (1971). An Introduction to Probability Theory and Its Applications.

[21] Levy M., Solomon S. (1996). Power laws are logarithmic Boltzmann laws. Int. J. Mod. Phys. C..

[22] Takayasu H., Sato A.H., Takayasu M. (1997). Stable Infinite Variance Fluctuations in Randomly Amplified Langevin Systems. Phys. Rev. Lett..

[23] Meeker W.Q., Hahn G.J., Escobar L.A. (2017). Statistical Intervals: A Guide for Practitioners and Researchers.

[24] Suzuki M., Oshima T. (1985). Co-ordination number of a multi-component randomly packed bed of spheres with size distribution. Powder Technol..

[25] Connelley M.S., Reipurth B., Tokunaga A.T. (2008). The Evolution of the Multiplicity of Embedded Protostars II: Binary Separation Distribution & Analysis. Astron. J..

[26] Rabeaud O., Royer J.J., Jébrak M., Cheilletz A. (2013). Log-uniform distribution of gold deposits along major Archean fault zones. Miner. Depos..

[27] Tsafrir D., Etsion Y., Feitelson D.G. (2005). Modeling User Runtime Estimates. Job Sched. Strateg. Parallel Process..

[28] Seven & i Holdings Co., Ltd. Corporate Outline 2011.

